# Extensive adipocytic maturation can be seen in myxoid liposarcomas treated with neoadjuvant doxorubicin and ifosfamide and pre-operative radiation therapy

**DOI:** 10.1186/2045-3329-2-25

**Published:** 2012-12-29

**Authors:** Wei-Lien Wang, Daniela Katz, Dejka M Araujo, Vinod Ravi, Joseph A Ludwig, Jonathan C Trent, Shreyaskumar R Patel, Patrick P Lin, Ashleigh Guadagnolo, Dolores Lòpez-Terrada, Angelo Paola dei Tos, Valerie O Lewis, Dina Lev, Raphael E Pollock, Gunar K Zagars, Robert S Benjamin, John E Madewell, Alexander J Lazar

**Affiliations:** 1 Department of Pathology, The University of Texas MD Anderson Cancer Center, 1515 Holcombe Blvd Unit 085, Houston, TX, 77030, USA; 2 Sharette Institute of Oncology, Hadassah-Hebrew University Medical Center, POB 12000, Jerusalem, 91120, Israel; 3 Department of Medical Oncology, The University of Texas MD Anderson Cancer Center, 1515 Holcombe Blvd Unit 085, Houston, TX, 77030, USA; 4 Sarcoma Multidisciplinary Program, The University Of Miami Sylvester Cancer Center, Room#C-050, 1475 Northwest 12th Avenue, Suite 3513, Miami, FL, 33136, USA; 5 Department of Othropeadic Surgical Oncology, The University of Texas MD Anderson Cancer Center, 1515 Holcombe Blvd Unit 085, Houston, TX, 77030, USA; 6 Department of Radiation Oncology, The University of Texas MD Anderson Cancer Center, 1515 Holcombe Blvd Unit 085, Houston, TX, 77030, USA; 7 Department of Pathology, Texas Children’s Hospital and Baylor College of Medicine, 6621 Fannin St, MC1-2261, Houston, TX, 77030, USA; 8 Department of Anatomic Pathology, General Hospital of Treviso, Piazza Ospedale 1, Treviso, 31100, Italy; 9 Department of Cancer Biology, The University of Texas MD Anderson Cancer Center, 1515 Holcombe Blvd Unit 085, Houston, TX, 77030, USA; 10 Sarcoma Research Center, The University of Texas MD Anderson Cancer Center, 1515 Holcombe Blvd Unit 085, Houston, TX, 77030, USA; 11 Department of Surgical Oncology, The University of Texas MD Anderson Cancer Center, 1515 Holcombe Blvd Unit 085, Houston, TX, 77030, USA; 12 Department of MusculoSkeletal Radiology, The University of Texas MD Anderson Cancer Center, 1515 Holcombe Blvd Unit 085, Houston, TX, 77030, USA

**Keywords:** Adipocytic maturation, Myxoid liposarcoma, Treatment

## Abstract

**Background:**

Trabectedin and thioglitazones have been documented to induce adipocytic maturation in myxoid liposarcoma; we have noted this in our experience as well. Intriguingly, we have also encountered this same phenomenon in myxoid liposarcomas exposed to various combinations of neoadjuvant doxorubicin and ifosfamide systemic chemotherapy with preoperative radiation, where the pathological effects have been less characterized. We examined the histological changes, including adipocytic maturation, associated with this treatment in patients with myxoid liposarcoma and evaluated for prognostic significance.

**Methods:**

Twenty-two patients were identified with histologically confirmed myxoid liposarcomas (9 with variable hypercellular areas) who were treated with neoadjuvant doxorubicin (75-90 mg/m2/continous infusion over 72h every 3 week) and ifosfamide (2.5 g/m2 daily x 4 every 3 weeks) for 4-6 cycles. Twenty-one patients received pre-operative radiation including 5 with concurrent gemcitabine. Pre- and post-treatment MRI studies were compared for changes in tumor area, fat content and contrast uptake, with the latter two estimated as: none, <25%, 25-49% and >50%. Post-treatment specimens were evaluated for hyalinization, necrosis and adipocytic maturation. Clinical follow-up was obtained.

**Results:**

Median age was 45 (26-72) years with a median tumor size of 11 (2-18) cm. All occurred in the lower extremities except for one case in the neck. As is common in myxoid liposarcoma, all had extensive treatment changes (>90%) with extensive hyalinization (n = 16; >90%) or prominent adipocytic maturation (n = 6; >50%) including 2 cases composed almost entirely of mature-appearing adipose tissue. Variable necrosis was identified (5-30%). MRI revealed a decrease in tumor area in all but one tumor (median, 65%), an increase in fat content in 7 tumors (n = 2, >50%;n = 2, 25-50%;n = 3,<25%), and a decrease in contrast enhancement in most tumors (n = 5, >50%; n = 9, 25-49%; n = 7, <25%). Median follow-up was 57 (12-96) months with 17 alive with no disease/metastases, 3 alive with disease and 2 dead of disease. Six patients developed metastases with median interval of 26 (22-51) months post resection. Four of 6 tumors with increased adipocytic maturation >50% on histology had increased fat detected by MRI (>25%). All 6 are alive but 2 developed metastases. In the remaining patients, 4 developed metastases with 14 alive and 2 dead of disease.

**Conclusion:**

Myxoid liposarcoma exposed to neoadjuvant doxorubicin and ifosfamide and pre-operative radiation can have prominent adipocytic maturation similar to trabectedin treatment. Myxoid liposarcomas exhibit extensive treatment changes with prominent hyalinization being the most common histological change. Despite this, patients develop metastases regardless of adipocytic maturation. While of unclear significance, no patient with fatty maturation died of disease.

## Background

Myxoid liposarcoma is the second most common malignant lipomatous tumor and preferentially affects ages younger than most other liposarcomas (young adults), and typically occurs in the lower extremities. Classically, these tumors are characterized by monotonous small short spindle cells in myxoid stroma with abundant thin-walled capillaries. Scattered lipoblasts, typically univacuolar, are variably present sometimes accompanied by variable amounts of more mature-appearing adipocytes [1,2]. Despite their banal histological appearance, metastases are common, and often to peculiar sites such as to the retroperitoneum and bone [3,4]. Tumors with increased hypercellular areas composed of enlarged, more round tumor cells without intervening myxoid stroma have an increased risk for metastases and poor prognosis [2-6]. Those with a significant proportion of round cell change are termed round cell liposarcoma and considered to be a higher grade variant of myxoid liposarcoma [7].

The vast majority of myxoid liposarcomas are characterized by a recurrent translocation involving t(12;16) (q13;p11) fusing the 3^′^-end promoter region of *FUS/TLS* (16p11) with the 5^′^-end of the *CHOP/DDIT3* locus (12q13) [8-14]. The resulting fusion protein is believed to play a critical role in tumorigenesis, blocking the PPARγ pathway which promotes adipocytic differentiation or maturation, offering insight into the primitive pre-adipocytic histological appearance of this tumor [15,16]. In rare situations, the alternative t(12;22) (q13;p11) resulting in a *EWSR1-CHOP/DDIT3* fusion transcript is present [9,17-20]. Other genetic mutations including involvement of the p53, RET, MET, and PI-3 kinase pathways, have also been implicated in myxoid liposarcomas [6,15,16,21-23].

**Figure 1:**
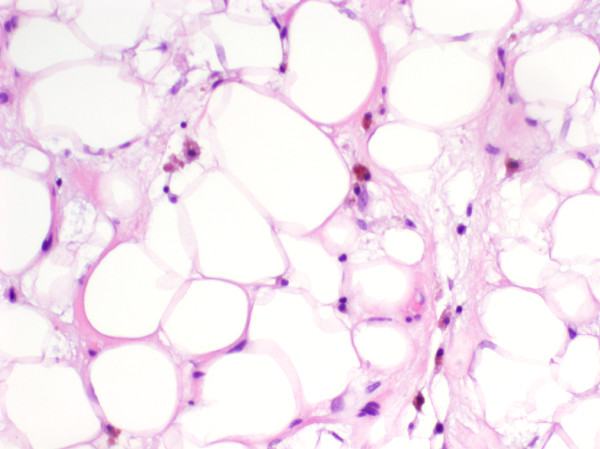
**Myxoid liposarcoma metastasis in a patient being treated with trabectedin.** Histological sections reveal irregularly-sized mature-appearing adipocytes in a myxoid stroma with thin-walled vessels. Adipocytic maturation is seen in patients receiving trabectedin therapy.

ET-743 (also known as trabectedin) is an alkaloid compound discovered from the sea squirt *Ecteinascidia turbinata* and found to show efficacy in the treatment of sarcoma patients including those with myxoid liposarcoma [24-27]. While the mechanism of action is not completely known, trabectedin may block transcription factor binding to the fusion gene *FUS-CHOP/DDIT3*[15]. It has also been well documented to induce differentiation in myxoid liposarcoma with prominent adipocytic maturation (Figure 1). In addition, PPARγ agonists, thioglitazones, have been reported to produce similar histological effects in myxoid liposarcomas [28]. These effects have been purported to be characteristic and perhaps specific and thus suggesting a particular mechanism of action leading to the release of a blockade of adipocytic maturation in the tumor.

We have encountered this phenomenon of adipocytic maturation in myxoid liposarcomas exposed to neoadjuvant doxorubicin and ifosfamide and pre-operative radiation, where the pathological effects have been less characterized. We examined the histological changes, including adipocytic maturation, associated with this treatment in patients with myxoid liposarcoma and evaluated for any prognostic significance of this feature.

## Methods

Under institutional board review approval, twenty-two patients were identified between 2000 and 2009 who had histologically confirmed (WLW, AJL) myxoid liposarcoma on pre-treatment biopsy and were not previously treated with chemotherapy. Nine of these patients had variable hypercellular/round cell change. Patients were treated with neoadjuvant doxorubicin (75-90 mg/m2/continous infusion over 72 hours every three weeks) and ifosfamide (2.5 g/m2 over three hours for four days, every three weeks) for 4-6 cycles. Twenty-one patients also received preoperative radiation including 5 with concurrent gemcitabine. Pre- and post-treatment MRI studies were compared (JM) for changes in tumor area, fat content and contrast uptake, with the latter two estimated as: none, <25%, 25-49% and >50%. Post-treatment specimens were compared with pre-treatment biopsies and evaluated for percentage of hyalinization, necrosis and adipocytic maturation (WLW, AJL). Clinical follow-up was obtained. Fluorescence in situ hybridization (FISH) on a selected post-treatment case for 12q13 to detect *CHOP/DDIT3* rearrangement was performed using the LSI *FUS* dual-color, break-apart probe (Abbott Molecular/Vysis, Des Plaines, IL, USA) according to the manufacturer’s recommendations as previously described [14].

## Results

Clinical, pathological and radiological findings are summarized in Table 1. The median age was 45 (26-72) years with a median size of 11 (2-18) cm. All occurred in the lower extremities except for one case in the neck.

**Table 1:** Summary of clinical, pathological and imaging factors

**Median age (range) years**	**45 (26-72)**
**Sex M:F**	13:9
**Location (n, %)**	
Thigh	15 (68%)
Calf	6 (27%)
Neck	1 (4.5%)
**Round Cell Component (n,range of %)**	n = 9 (5-30%)
**Follow-up**	
Median, range	57 mos (12-96 mos)
Alive and Well (median, range)	n = 17 (35 mos, 12-96 mos)
Alive with Disease (range)	n = 3 (61,67, 72 mos)
Dead of Disease (range)	n = 2 (82, 16 mos)
Metastases (median, range)	n = 6 (26, 22-51 mos)
**Post-Treatment Pathological Findings**	
***Median Size (range)***	11 (2-18) cm
***Tumor with Treatment Changes (median %, range)***	95% (90-95%)
***Hyalinization (median %, range)***	90% (10-95%)
***Fat Maturation***	
Median (range)%	10% (5-90%)
Prominent >50% (n)	n = 6 with 2 >90%
***Necrosis (median %, range)***	5% (5-10%)
**Radiological Changes**	
***Decrease in Tumor Size*** (median % decrease, range)	65% (30 - 94%)*
***Fat Amount Change (n, % of cases with changes)***	
Decrease <25%	1 (4.5%)
No change	14 (63.6%)
Increase <25%	3 (13.6%)
Increase 25-49%	2 (9.1%)
Increase >50%	2 (9.1%)
***Contrast Media Change (n, % of cases with changes)***	
Increase <25%	1 (4.5%)
Decrease <25%	7 (31.8%)
Decrease 25-49%	9 (40.9%)
Decrease >50%	5 (22.7%)

**Figure 2:**
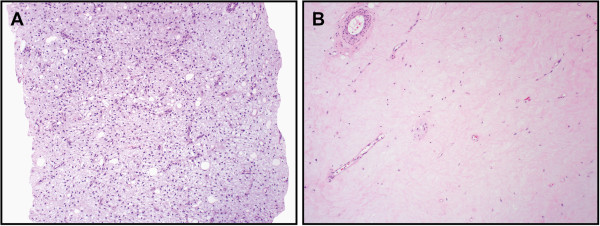
**(A) Pre-treatment biopsy reveals characteristic bland spindle cells in myxoid stroma with branching thin walled vessels.** (**B**) Post treatment specimen reveals extensive hyalinization that is essentially acellular. This was the most common pattern seen in treated tumors.

**Figure 3:**
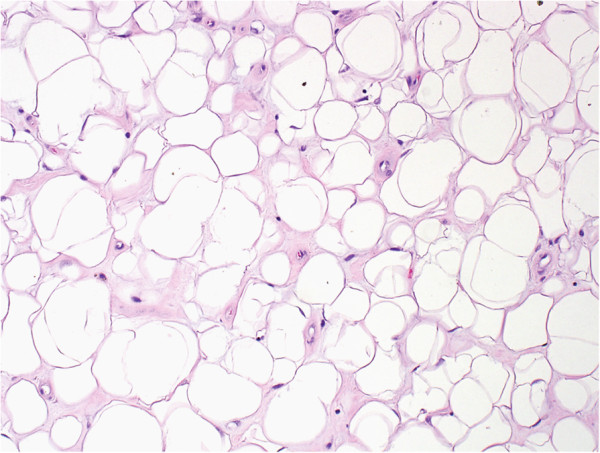
**Histological example of extensive fatty maturation with variable sized adipocytes.** Extensive fatty maturation was seen in one-third of cases; two of which had adipocytic maturation that was greater than 90%.

All developed extensive post-treatment changes (>90%) with extensive hyalinization (>90%) being the most common change seen (16/22 patients, 72%) (Figure 2). Focal residual areas of characteristic vascular pattern were often identified. Prominent fatty maturation (>50%) was seen in 6/22 (27%) cases including 2 cases composed almost entirely of mature-appearing fat (Figure 3). Both cases received radiation therapy; one also received gemcitabine. One tumor with extensive post-treatment fatty maturation was tested and found to retain the rearrangement in *CHOP/DDIT3* verifying that the mature adipocytes arose from the tumor cells. Variable necrosis was also identified (5-30%).

**Figure 4:**
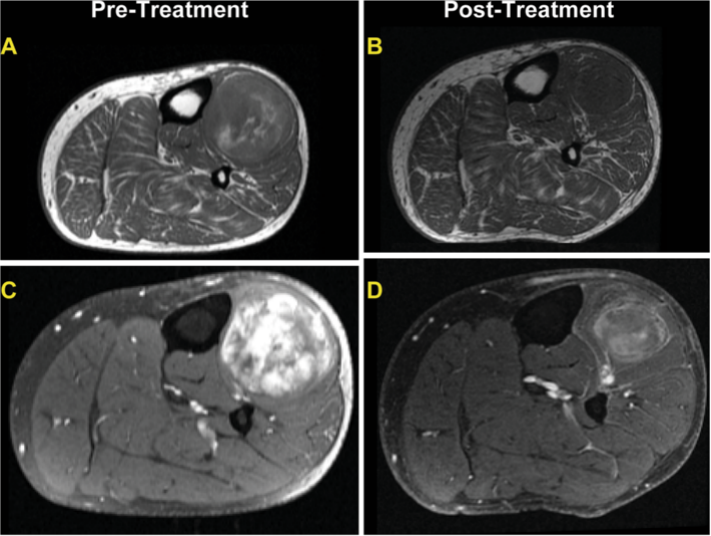
**Example of MRI findings seen in the majority of myxoid liposarcomas undergoing treatment in our series.** T1 weighted axial image of the calf with mass (**A**) pre-treatment and (**B**) post treatment showing a reduction in tumor size and decreased adipocytic content. T1 weighted post contrast media with fat saturation with (**C**) pre-treatment demonstrating an extensive heterogeneous enhancement, while (**D**) post treatment shows decreased tumor size and decreased enhancement greater than 50%. These findings correspond to increased hyalinization and decreased vascularity histologically.

**Figure 5:**
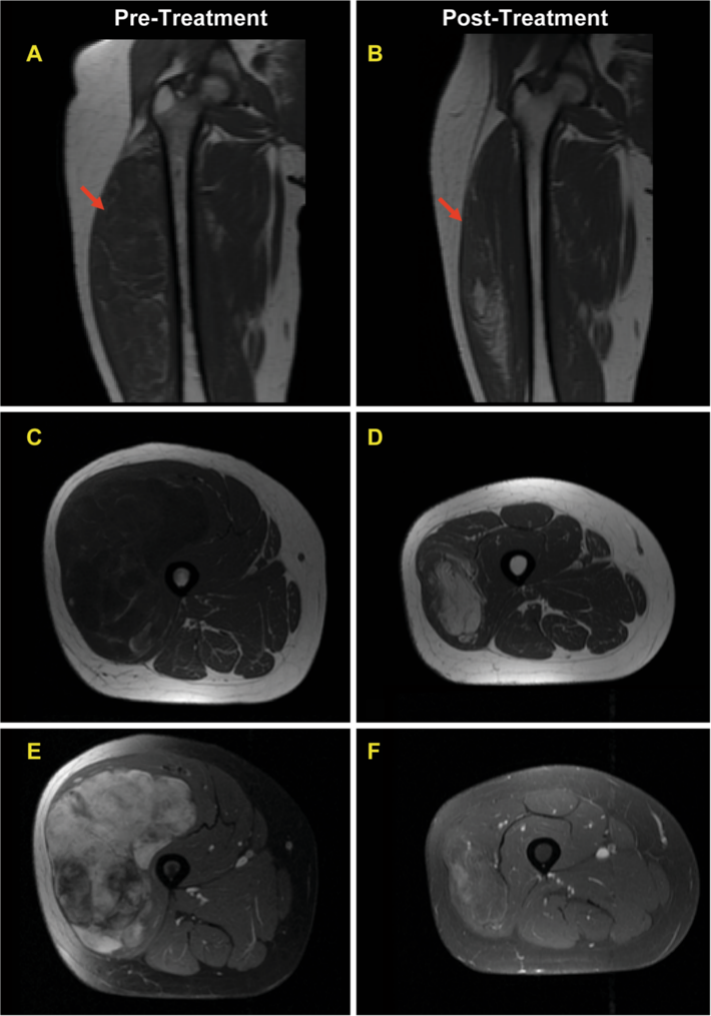
**MRI findings in a case of myxoid liposarcoma which developed extensive adipocytic maturation during treatment.** T1 weighted coronal right thigh (**A**) pre-treatment and (**B**) post treatment demonstrating a marked decrease in tumor size. T1 weighted axial image of right thigh (**C**) pre-treatment and (**D**) post treatment demonstrate an increase in signal characteristic of mature adipose tissue content (>50%) with reduction in size. T1 weighted axial with contrast media and fat saturation (**E**) pre-treatment showing large mass with extensive vascular enhancement. (**F**) Post treatment scans show a reduction in size of mass and a decrease in vascular enhancement greater than 50%.

MRI revealed a decrease in tumor area in all but one tumor (median decrease in size was 65%). A decrease in contrast enhancement was found in most tumors (n = 5,>50%; n = 9, 25-49%; n = 7, <25%) (Figure 4), histologically corresponding to increased hyalinization, decreased cellularity and decreased vasculature. Seven tumors developed an increase in a register characteristic of mature adipose tissue (n = 2,>50%; n = 2, 25-49%; n = 3,<25%) (Figure 5). Four of 6 tumors with histological adipocytic maturation >50% also had increased adipose signal detected by MRI (>25%).

Median follow-up was 57 (12-96) months with 17 patients alive with no disease/metastases, 3 patients alive with disease and 2 patients dead of disease. Six patients developed metastases with a median interval of 26 (22-51) months post resection. All 6 patients with adipocytic maturation are alive although 2 developed metastases.

## Conclusions

Myxoid liposarcomas treated with neoadjuvant doxorubicin and ifosfamide and pre-operative radiation consistently develop extensive treatment effect that can include adipocytic maturation as demonstrated on MRI imaging and confirmed histologically at resection. In our series, the most common treatment change was extensive hyalinization (16/22, 72%) with decreased vasculature as seen in other studies [29]. In addition, approximately one-third of our patients also developed prominent adipocytic maturation, comprising more than 50% of the treated tumor. Two tumors had extensive adipocytic differentiation giving a misleading appearance of a well-differentiated liposarcoma or even a benign lipoma. Fortunately, most tumors also harbored areas of hyalinization, characteristic of treated myxoid liposarcoma. They also lacked the fibrous bands with scattered pleomorphic cells seen in most well-differentiated liposarcomas. Molecular testing, either for rearrangements involving *CHOP/DDIT3* by fluorescence in-situ hybridization assay or reverse polymerase chain reaction for the specific fusion transcripts, can also be helpful in confirming the diagnosis of myxoid liposarcoma [14,30]. In our study, rearrangement was still identified in tumors with prominent fatty differentiation by fluorescence in-situ hybridization assay. We chose this modality since it allows one to determine the cellular component of the tissue showing the re-arrangement. Well-differentiated liposarcomas would lack this rearrangement and would instead demonstrate amplification of the 12q13-15 region [31].

The phenomenon of adipocytic maturation is comparable to myxoid liposarcomas in patients treated with trabectedin and the thioglitazone family of PPARγ agonists. In 2007, Grosso and colleagues described the development of “mature lipoblast-featuring cells” and “clear adipocytic differentiation” in myxoid liposarcomas treated with trabectedin [24]. Gronchi and colleagues reported maturation to lipoblasts, decreased cellularity and vascularity in the tumors of 16 of 23 patients who received neoadjuvant trabectedin for advanced localized myxoid liposarcomas [32]. Forni and colleagues saw similar findings in myxoid liposarcoma cell lines and two patients treated with trabectedin [33]. Similarly, Demetri and colleagues also saw adipocytic differentiation as early as six weeks in tumors from three patients treated with troglitazone, a PPARγ agonist. These changes coincided with an increased expression in mRNA associated with adipocytic differentiation including AP2, adipsin and PPARγ. Mild increase in adipose density signaling was also seen in these tumors on MRI imaging [28]. In our series, extensive treatment changes, predominantly hyalinization with decreased cellularity and vascularity, was seen in the tumors of our patients treated with doxorubicin and ifosfamide with pre-operative radiation therapy. However, adipocytic maturation was also seen in our tumors, histologically indistinguishable to what has been reported previously reported by others and also seen by us in myxoid liposarcoma tumors treated with trabectedin (WLW, AJL, PT). In most cases, MRI imaging correlated with evolution of treatment changes seen in the resection specimen. Decreased vascularity by contrast media enhanced imaging was seen in the majority of cases with extensive hyalinization and subsequent decrease of the thin-walled vessels in these areas. In addition, all four cases that had an increase of fat (>25%) by MRI also showed prominent adipocytic maturation on histological resection.

Myxoid liposarcoma can rarely have areas with mature-appearing adipocytes as well. Evans reported 29 cases of myxoid liposarcoma, some of which had areas that were reported to resemble mature, non-neoplastic adult adipose tissue [1]. In a more recent study by Fritchie and colleagues, 10 of 46 (22%) myxoid liposarcomas had a mature fat appearing lipomatous component that composed 5-79% of the tumor. Full information regarding treatment was not provided in either study. Some degree of stromal hyalinization was also observed in seven cases in the Fritchie study, six of which were known to be untreated. However, this was only focal, composing only 1-9% of these tumors, in contrast to our treated cases which had extensive hyalinization [7]. It is possible that some changes could have been present before treatment and not sampled in the pre-treatment biopsy, emphasizing the importance of comparing pre- and post-treatment imaging studies. One case with histological evidence of adipocytic maturation revealed no change in adipocytic content by MRI and adipocytic maturation was noted in the pre-treatment biopsy. However, in our experience (WLW, AJL) extensive fatty maturation and hyalinization are uncommon in untreated myxoid liposarcomas. The remaining pre-treatment biopsies lacked these changes and developed increased fat on MRI studies between pre- and post treatment tumors, which corresponded histologically to increased fatty maturation, increased hyalinization and decrease vascularity on the resection specimen.

The significance of adipocytic maturation is unclear. No patients with extensive adipocytic maturation died of disease, but patients did develop metastases regardless of adipocytic maturation. However, our series is small and additional larger studies are necessary. The mechanism of fatty maturation when exposed to doxorubicin and ifosfamide and radiation therapy is also not known. Doxorubicin is an anthracycline and proposed to have many mechanisms of actions among which include intercalcating DNA, formation of free radicals, inhibition of topoisomerase II, prohibiting DNA and RNA unwinding, and induction of cell death [34]. Ifosfamide is a DNA alkylating agent which prohibits DNA replication and results in cell death [35]. Interestingly, doxorubicin at low concentrations has been reported to induce differentiation in leukemia and breast carcinoma cell lines [34,36,37]. In breast cell lines, differentiation corresponded to the reduction in c-myc expression and growth arrest in response to doxorubicin induced DNA damage [37]. To our knowledge, ifosfamide has not been reported to induce differentiation. Perhaps a similar non-specific response to DNA damage with doxorubicin can occur in myxoid liposarcomas. Other sarcomas have also been reported to differentiate in response to chemotherapy and radiotherapy including rhabdomyosarcoma [38]. Our study would support the notion that these changes can be a general response to effective treatment in myxoid liposarcoma and may not necessarily be specific to a treatment modality such as trabectedin. In this context, we should be cautious in interpreting potential insights into the unknown mechanism of action of trabectedin from this phenomenon.

The fusion transcript characteristic in the vast majority of myxoid liposarcomas is composed of *FUS,* which encodes for a RNA/DNA binding domain, and *CHOP/DDIT3,* which encodes for a transcription factor which dimerizes with CAAT/enhancer binding proteins (C/EBP) and negatively regulates genes involved in adipocytic differentiation including PPARγ. Multiple fusion variants and types exist; however, none have been associated with increased adipogenesis [39]. Trabectedin binds to the minor groove of DNA and is theorized to sterically hinder the binding of the FUS-CHOP/DDIT3 fusion protein (probably mediated primarily by the CHOP/DDIT3-donated DNA binding domain) and thus allow adipocytic differentiation to continue by relieving repression. It could also effect the post transcriptional modification of the fusion transcript [15]. In addition, other mutations are known to exist including activation of the ERK/MAPK, PI-3-kinase/Akt pathways, alterations of p53, IGF1R and IGF2; some of these have been associated with round cell change and poor prognosis. [16,22] None are known to correlate with adipocytic differentiation.

In summary, we report the histological and radiological findings seen in myxoid liposarcomas in patients treated with doxorubicin and ifosfamide and pre-operative radiation therapy. The vast majority of these tumors demonstrated significant treatment effect in the form of hyalinization; however, adipocytic maturation, once thought to be exclusive to tumors treated with trabectedin and PPARγ agonists, were also seen in some of our cases. Pathologists and radiologists should be aware of this as it may cause diagnostic confusion if prior history is not available and in comparing histological treatment changes between trials.

## Competing interests

The authors declare that they have no competing interests.

## Authors’ contributions

WLW reviewed the histological findings and wrote the publication; AJL reviewed the histological findings and is the senior author on this publication; PT reviewed selected histological findings; JM reviewed the radiological studies; DL edited the manuscript; VOL, PL, and RP surgically removed the specimens; AG and GZ performed the radiation treatment; RB, SP, RV, DA, and DK administered the chemotherapy, DLT performed the fluorescence in-situ hybridization. All authors read and approved the final manuscript.
